# Early allergen introduction overrides allergy predisposition in offspring of horses with *Culicoides* hypersensitivity

**DOI:** 10.3389/fimmu.2025.1654693

**Published:** 2025-10-21

**Authors:** Elisabeth M. Simonin, Sigurbjorg Torsteinsdóttir, Vilhjálmur Svansson, Sigríður Björnsdóttir, Heather Freer, Justine Tarsillo, Bettina Wagner

**Affiliations:** ^1^ Department of Population Medicine and Diagnostic Sciences, College of Veterinary Medicine, Cornell University, Ithaca, NY, United States; ^2^ The Institute for Experimental Pathology, at Keldur, University of Iceland, Reykjavik, Iceland; ^3^ Office of Animal Health and Welfare, Icelandic Food, and Veterinary Authority, MAST (MAST), Selfoss, Iceland

**Keywords:** equine, allergen introduction, allergy development, full-sibling, allergen-specific IgE, allergy

## Abstract

**Introduction:**

The origins of allergy are both genetic and environmental. We performed a full-sibling study to determine the role of early-in-life or delayed allergen introduction on *Culicoides* hypersensitivity development in a cohort with history of an allergic phenotype and Culicoides hypersensitivity. IgE-mediated allergies naturally develop in many mammalian species, and we used a horse model of allergy called *Culicoides* hypersensitivity. *Culicoides* hypersensitivity is a seasonal, recurrent, IgE-mediated allergy caused by the salivary proteins of biting *Culicoides* midges.

**Methods:**

The study included four cohorts that lived together in the same environment, only differing in the timing of allergen exposure and the transfer of allergen-specific maternal antibodies. The parent cohort was first exposed to allergens in adulthood, and each full-sibling cohort was first exposed to allergen either in puberty or at birth. All full-siblings had at least one allergic parent with an allergic phenotype, suggesting a predisposition to develop allergy. Allergen-specific IgE and IgG isotypes were measured before and after exposure to *Culicoides* to determine whether maternal-acquired allergen-specific antibodies influenced the rate of *Culicoides* hypersensitivity development. All four cohorts were followed for at least nine years of allergen exposure.

**Results:**

The rate of allergy development was inversely related to the timing of allergen exposure where introduction in adulthood led to the highest rate of allergy development (62.5%), a moderate allergy rate was found for introduction during adolescence (21.4%), and no individuals exposed at birth developed *Culicoides* hypersensitivity. In addition, exposure to maternally-acquired allergen-specific IgE and IgG did not influence the rate of allergy development in the cohorts exposed to allergen at birth.

**Discussion:**

We provide strong evidence in a full-sibling study that early-in-life allergen exposure, independent of maternal allergen-specific immunoglobulin, prevents *Culicoides* hypersensitivity development in individuals born to parents with an allergic phenotype.

## 1 Introduction

The prevalence of allergies is steadily increasing in humans and animals, such as dogs, cats, and horses. As a result, there is urgent focus to determine what predisposes an individual to develop allergies, and whether allergies can, therefore, be prevented.

It is accepted that both genetic and environmental factors increase the risk of allergy. In fact, in humans, children with one allergic parent are twice as likely to develop the same allergy, and three times as likely if both parents are allergic (1). Polymorphisms in MHCII HLA genotypes DR and DQ are linked to peanut, cow’s milk and egg allergies (2, 3), and risk alleles in the genes for MALT1 and filaggrin are associated with increased risk for food allergy and atopic dermatitis, respectively (4, 5). However, while these associations support the heritability of food allergy (6), they do not account for the different timing of allergen introduction to individuals. Families with allergic members tend to eliminate allergens from the entire family’s diet. As a result, while genetic factors may promote the risk of food allergy, they are also often combined with adjusted behavior, like delayed or absent introduction to allergen (7).

The timing and frequency of allergen introduction has, therefore, been suggested to be an important risk factor. There is no available data or studies in horses that address this research question. Therefore, this study was designed to address the lack of data in horses. However, we can gain some insight from human allergy studies. In humans, early allergen exposure in the first year of life decreased the risk of allergy development, even in children with allergic parents (5, 8, 9). Together, these studies supported that early introduction to allergy is important in preventing allergy.

However, while these associations in humans are valuable and convincing, there are still limitations including recall bias, differences in allergen consumption such as concentration, frequency and timing by both mother and child, and unrealized accidental allergen exposures. Due to these variables inherent in human cohorts, and the lack of full control over an individual’s exposure to allergen, many studies present contradicting results on the protective or worsening effect of early allergen introduction. For example, four independent studies to prevent egg allergy determined that early egg introduction both did (8, 9), and did not (10, 11), prevent allergy development. This highlights the urgent need for additional allergy models to parse the role of early-in-life allergen introduction on allergy development. Controlled experiments in animals that naturally develop IgE-mediated allergies, like dogs, cats or horses, can be performed to remove confounders typically associated with human studies.

Horses can develop an IgE-mediated allergy called *Culicoides (Cul)* hypersensitivity, also known as summer eczema, insect bite hypersensitivity or sweet itch (12–17). The equine disease is mechanistically comparable to IgE-mediated allergies in humans (13, 18, 19), and specific genetic risk factors, like MHCII gene variants associated with an increased risk for allergy development, have been identified (20, 21). *Cul* hypersensitivity is a seasonal, recurrent skin allergy with clinical signs of pruritus, alopecia and dermatitis. The responsible allergens are salivary proteins of *Cul* midges which are injected into the horse’s skin with frequent midge’s bites during the summer (17, 18, 22–30). *Cul* midges live in most regions of the world. However, in Iceland, *Cul* did not reside until 2015. Interestingly, epigenetic studies have found that horses showed the highest prevalence of *Cul* hypersensitivity, ranging from 50-82%, when imported from Iceland to Europe as adults and first exposure to *Cul* allergens occurred during adulthood (31–33). In contrast, Icelandic horses born in Europe had lower *Cul* hypersensitivity prevalence, ranging from 8.2-12.6% (32, 34). However, the specific cause of these different allergy rates has not yet been studied.

Based on the evidence in human allergies that there is a higher risk of allergy in children born to allergic parents (6), maternally-acquired allergen-specific immunoglobulins may also have a role in allergy development. This is not possible to study in humans as many immunoglobulins can cross the placenta during gestation (35). However, in horses, maternal immunity transfer, including maternal IgE and IgG, occurs exclusively after birth with the colostrum (36). Therefore, a horse model of *Cul* hypersensitivity allows the unique opportunity to evaluate the role of early allergen-specific immunoglobulin exposure in the acceleration or prevention of allergy development.

Together, these prior studies led to the two hypotheses that (1) timing of introduction, in adulthood versus early-in-life, and (2) maternal allergen-specific IgE exposure are dominant determinants for allergy development. Here, we conducted a full-sibling longitudinal study using a horse model of clinical *Cul* hypersensitivity. Our model used Icelandic horses that were completely naïve to *Cul* allergen when they were exported to the United States (US) in 2012 or 2013 as adults or at teenage. Two additional full-sibling cohorts were born in the US and exposed to allergen starting at birth. One of these cohorts received allergen-specific maternal IgE from the dams, and the other did not. This study offered the unique opportunity to control the timing of first allergen introduction and subsequent annual allergen exposure, as well as maternally-acquired allergen-specific immunoglobulin transfer, in the parent and three full-sibling cohorts while monitoring the development of allergy.

This study targeted to identify the influence of early allergen exposure and/or maternal allergen-specific IgE exposure on the development of an allergic phenotype caused by *Culicoides* midges. Overall, the present study identified the impact of early allergen introduction and maternal allergen-specific IgE transfer in a controlled full-sibling model of clinical IgE-mediated allergy with a high genetic risk for allergy development.

## 2 Materials and methods

### 2.1 Animals

This study followed three full-sibling cohorts of offspring (n=15 per cohort), and their parents (15 mares, one stallion), before, during and after allergen introduction. All individuals were Icelandic horses and either remained clinically healthy or developed clinical allergy. The full-sibling study allowed a close genetic relationship between cohorts while still maintaining a heterogenetic gene pool. The parents had high levels of MHC heterozygosity and haplotype diversity, as described in a prior study using the same cohorts of horses (which included all mares, stallion and all three full-sibling cohorts) (37).

All 61 horses were on the same diet, grazing in the summer and fed grass hay in the winter. In addition, the full-sibling foals freely suckled colostrum and milk from their dams until weaned at eight months of age. Horses were kept full time on large pastures with run-in-sheds, free access to water, and mineral salt blocks. Vaccination and deworming of all horses were synchronized: each horse was annually vaccinated against rabies, tetanus, West Nile virus, and Eastern and Western Encephalitis virus, as well as dewormed with moxidectin and praziquantel (Zoetis, Parsipanny, NJ, USA) once a year in December. Foals were first vaccinated at eight months of age and then were annually vaccinated. The horses did not receive any other treatments.

All animal procedures were carried out in accordance with the recommendation in the Guide for the Care and Use of Laboratory Animals of the National Institutes of Health. The animal protocol was approved by the Institutional Animal Care and Use Committee at Cornell University (protocol #2011–0011). The study also followed the Guide for Care and Use of Animals in Agricultural Research and Teaching.

### 2.2 Environment and allergen exposure

All horses lived together in the same two environments, either in Iceland (Reykjavik or Hólar) without allergen exposure (parents and full-sibling cohort 1), and/or in the US (Ithaca, NY) with allergen exposure (parents and full-sibling cohorts 1-3). Horses were not exposed to any *Cul* midge allergens while in Iceland or during winter months in Ithaca. Daily minimum and maximum temperatures were recorded for 2012–2016 from wunderground.com at a weather station <1 mile from the horse pastures.

### 2.3 Full-sibling cohorts

**Figure 1:**
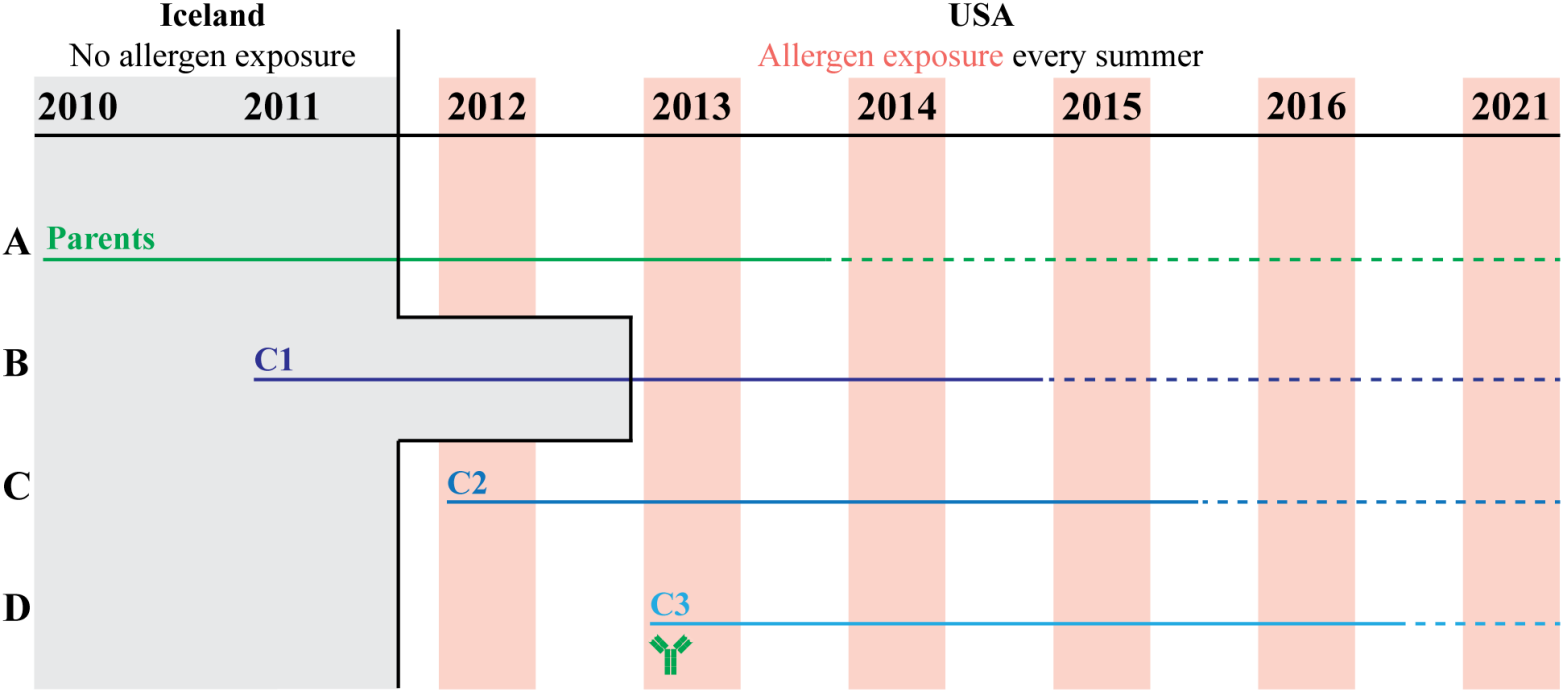
Study timeline of allergen exposure and allergen-specific immunity transfer from dams to foals. *Culicoides* (*Cul*) hypersensitivity is an IgE mediated allergy and clinical signs recur every summer when Cul midges are in the environment. A group of 15 mares were bred to the same stallion three consecutive years. The three full sibling generations experienced different timing of allergen introduction and exposure (C1 in puberty versus C2 & C3 from birth-on), and different timing of allergen specific immunity transfer from the dams (C1 & C2 received no allergen-specific maternal immunity; C3 had maternal allergen-specific immunity transfer at birth), while maintaining a close genetic relationship. *Cul* allergen was not present at any time in Iceland (gray shaded section) and was only present during warm summer months in the USA (pink shaded sections). Each group remained in the same environment of allergen exposure (solid horizontal lines) for at least 2 summers (Parents & C1) or 4 summers (C2 & C3). Afterwards, individuals either stayed in this environment (Cornell) or moved to different locations in the US (allergen endemic). A follow up survey was conducted in 2021 to confirm the allergy status of all horses (dotted horizontal lines). **(A)** The parent generation (green line) was first exposed to allergen in the summer 2012 as adults. **(B)** C1 (dark blue line) was first exposed to allergen in the summer 2013 at 2 years of age. **(C)** C2 (medium blue line) was first exposed to allergen at birth and was born to dams that had not yet become sensitized to allergens. **(D)** C3 (light blue line) was first exposed to allergen at birth. Their dams had been exposed to allergen the year prior, developed clinical allergy that summer or stayed clinically healthy, and developed allergen-specific IgE which was transferred to the C3 foals with the colostrum (green antibody).

This study followed the development of seasonal, recurrent *Cul* hypersensitivity in three full-sibling cohorts and their parents as summarized in **Figure 1**, **Table 1** and **S5**. Briefly, allergen exposure occurred only when cohorts were living in the US and then occurred annually during warm summer months. The parents were born and lived in Iceland until February 2012, when they were imported as adults to the US, and were first exposed to *Cul* midges on May 15, 2012. Each mare foaled three consecutive full-sibling foals from the same stallion. Full-sibling cohort 1 (C1) was conceived in Iceland in 2010, born in May-June 2011, imported to the US in April 2013, and first exposed to *Cul* in May 2013 at 2 years of age (adolescence for horses). Cohort 2 (C2) was conceived in Iceland in 2011, imported to the US *in utero* in February 2012, and born in June 2012. Cohort 3 (C3) was conceived in the US in 2012 and born in June 2013. C2 and C3 foals were exposed to *Cul* from birth-on.

**Table 1:** Timing of allergen introduction, maternal immunity, and rate of allergy in each cohort.

Cohort	Sex	Age (years) at allergen introduction^†^ median (range)	Maternal allergen-specific immunity^‡^	Age at follow-up	Rate of allergy^§^ (% allergic)	Rate of allergy, p value^¶^
Parents	Male (n=1) Female (n=15)	Median: 16 Range: 8 (5-13)	no	Median: 25 Range: 17 (14-22)	1 allergic (100%) 9 allergic (60%)	–
C1	Male (n=9) ^#^ Female (n=5)	2	no	10	2 allergic (22%) 1 allergic (20%)	0.0146
C2	Male (n=9) Female (n=6)	At birth	no	9	0 allergic (0%)	<0.0001
C3	Male (n=6) Female (n=9)	At birth	yes	8	0 allergic (0%)	<0.0001

† Parents were exposed to allergen, upon export from Iceland, as adults. Full-sibling C1 was exposed to allergen, upon export from Iceland, in adolescence. Full-sibling C2 & C3 were born in the US and exposed to allergen from birth-on.

‡ The presence of Cul o 2 and Cul o 3-specific IgE in colostrum was measured by ELISA.

§ Clinical allergy was quantified by weekly to biweekly allergy scoring based on pruritus, alopecia, and dermatitis. Allergic horses had monthly average scores ≥3 for the entire summer, and clinical disease recurred in each subsequent summer.

¶Statistics compare rate of allergy in C1–3 with the Parent cohort. Statistics were run on entire cohort including both male and female individuals.

#One C1 horse (n=1, male) died in October 2013 at age 2.5 years after only one summer of allergen exposure. This foal did not develop allergy and is excluded from all analyses due to the lack of a complete follow up.

### 2.4 Allergy development and scoring

Clinical allergy was observed in allergic animals from the end of May to early October. *Cul* hypersensitivity was confirmed, as previously described (14, 19), by intradermal skin testing with allergens, including *Cul* whole body extract (WBE; Stallergenes Greer Inc., Cambridge, MA, USA), in comparison to injections with saline and histamine as negative and positive controls, respectively. Allergic horses developed immediate reactions to *Cul* WBE, while healthy horses did not.

Clinical allergy scores were assigned to quantify the clinical signs of allergy for each horse, as previously described using the Cornell Scoring System formerly described as Scoring System A by Miller et al. (26). Scores were assigned on a scale of 0–10 based on pruritus (0-3), alopecia (0-4), and dermatitis (0-3). Scores were assigned 2–3 times per month while *Cul* was absent (December - March), and 3–5 times per month when *Cul* was present in the environment (April-November). Mean monthly allergy scores were calculated for each horse every year. Horses that developed persistent and recurrent clinical allergy with a mean monthly clinical score ≥3 over two or more months and also that recurred for multiple subsequent years were considered allergic. *Cul* hypersensitivity was defined as pruritus, alopecia and dermatitis that is persistent during *Cul* allergen exposure and also recurrent during each season of *Cul* exposure.

Horses that were born to parents that developed *Cul* hypersensitivity were also considered to have a genetic predisposition to develop *Cul* hypersensitivity based on the allergic phenotype of their parents. While not specifically characterized, we refer to this genetic background as an “allergic phenotype”.

### 2.5 Allergy follow-up survey

Starting at 3.5 years of age, several of the clinically healthy full-siblings left the Ithaca herd for private owners living in different geographic locations across the US. In addition, some clinically healthy parents left the herd after living in Ithaca for two years or longer. All horses remained in *Cul* endemic locations. We performed a follow-up owner survey of all horses in the summer 2021. We received responses on 56 out of 60 horses that were still alive from the study.

Horse owners were contacted by email or phone and asked to voluntarily complete a survey for each horse they owned from the study (**S3**). The survey results were used to adjust the allergy status if a horse developed allergy after leaving the Ithaca herd. The first set of survey questions (Questions 1-4) inquired about clinical signs (onset, severity, recurrency, seasonality), housing circumstances (inside/outside), and geographic location. The second set of questions, Questions 5-9, were only asked if the horse owner reported clinical signs of allergy on Question 4. Vague survey responses were followed up by email asking the owner additional questions to ensure that allergy status was accurately recorded.

### 2.6 Allergen-specific IgE and IgG in colostrum and serum

Allergen-specific immunoglobulin was measured in colostrum and foal serum by ELISA or fluorescent bead-based assay as previously described (16, 38), with some modifications: Serum was collected from foals at day 0 before suckling (d0), day 5 (d5), day 25-28 (1m) and 3 months (3m) by venipuncture of the *V. jugularis* using the BD vacutainer system (Becton Dickinson, Franklin Lakes, NJ). Serum was stored at -20°C. Colostrum was collected from dams before foals suckled, within 2 hours of birth, and stored at -20°C. Colostrum from one mare of a C3 foal was not collected. Upon thawing, colostrum was centrifuged at 2200xg, 5 min, room temperature to separate the fat. The lower hydrophilic phase was collected for antibody testing by ELISA or fluorescent bead-based assay.

*Culicoides obsoletus* allergens Cul o 2 (KC339672) and Cul o 3 (KC339673) were previously determined to be the dominant allergens in the parent cohort (16) and were used to measure allergen-specific IgE and IgG responses in this study. Both Cul o 2 and Cul o 3 were expressed in *P. pastoris* and were purified by fast protein liquid chromatography.

Allergen-specific IgE was measured by ELISA using well-validated monoclonal antibodies (39). Microtiter plates (Nunc, ThermoFisher Scientific, Waltham, MA, USA) were coated at 4°C overnight with 4 µg/ml Cul o 2 or Cul o 3 allergens in 100 µl carbonate buffer (1M NaHCO_3_, 1M Na_2_CO_3_, pH 9.6). Wells were washed with PBST and then incubated with serum or colostrum diluted 1:10 in PBST (PBS, supplemented with 0.05% Tween20). Serum from an allergic mare was run as a standard curve in four serial 1:2 dilutions and a starting dilution at 1:10. Following room temperature incubation and another wash, a biotinylated monoclonal antibody (mAb) against equine IgE (clone 134 (38), diluted to 2 µg/ml in PBST) was added and incubated at room temperature. Plates were again washed, then incubated with peroxidase-conjugated streptavidin (Jackson ImmunoResearch, West Grove, PA, USA), followed by substrate solution. Well absorbance was measured on a plate reader. Relative antibody units (RU) were calculated based on the standard curve which quantified *Cul-*specific IgE within the range of 10–100 RU. Absorbance values that were less than the plate blank were considered 0 RU. Absorbance values that were between the blank and the lowest standard value were considered 5 RU. A few samples in C3 had *Cul-*specific IgE absorbances above the maximum standard curve and were reported as 110 RU.

Allergen-specific IgG1 and IgG3/5 were measured in a multiplexed fluorescent bead-based assay (Luminex Corp., Austin, TX, USA), as previously described (16) using IgG isotype-specific monoclonal antibodies (39). A total of 5x10^6^ fluorescent microsphere beads (Luminex Corp., Austin, TX, USA) were coupled with 100µg of Cul o 2 or Cul o 3 allergen following the previously described protocol (40). Cul o 2 was coupled to fluorescent bead number 40 and Cul o 3 was coupled to fluorescent bead number 39. Millipore Multiscreen plates (Millipore, Danvers, MA, USA) were incubated with PBN (PBS, supplemented with 1% BSA and 0.05% sodium azide, pH 7.4) for 5 min. The buffer was removed through vacuum aspiration using an EL X 50 plate washer (Biotek Instruments Inc, Winooski, VT, USA). Colostrum or serum samples were added to the wells and mixed with a total of 5x10^3^ Cul o 2-coupled beads and 5x10^3^ Cul o 3-coupled beads. All serum samples were run 1:10, and all colostrum samples were diluted 1:50 in PBN before addition to the assay plate. Serum from an allergic mare was run as a standard curve in six serial 1:5 dilutions that started at 1:10. Samples were incubated together with allergen-coupled beads on a shaker at room temperature and in the dark for 30 minutes. After washing with PBST, biotinylated mAbs against equine IgG1 (clone CVS45 (41), diluted to 4 µg/ml in PBN) or IgG3/5 (clone 586 (42), diluted to 4 µg/ml in PBN) were added and incubated for 30 min on the shaker, in the dark at room temperature. After this step, plates were washed again with PBST and incubated with Streptavidin-phycoerythrin (Invitrogen, Carlsbad, CA, USA) diluted 1:100 in PBN for 30 min on the shaker, at room temperature, in the dark. Following a final PBST wash, beads were resuspended in 100µl PBN, incubated on the shaker for 15 minutes and analyzed with a Luminex 200 instrument (Luminex Corp., Austin, TX, USA). Allergen-specific IgG1 and IgG3/5 was measured as median fluorescence intensity of phycoerythrin (MFI).

### 2.7 Statistical analysis

D’Agostino-Pearson normality tests were used to determine that most of the cohort clinical score datasets were not normally distributed. As a result, nonparametric approaches were used for all analyses. To compare rate of allergy between cohorts, a Kruskal-Wallis test with Dunn’s multiple comparisons test was run. To compare clinical signs of allergy between allergic and nonallergic groups in each cohort and at different timepoints and to compare allergen-specific IgE, IgG1 and IgG3/5 in colostrum and foal serum, a non-parametric Tukey’s multiple comparisons test was used. All graphs plot median and range unless specified otherwise. *p* values <0.05 were considered significant. Analysis was performed and graphs were generated with GraphPad Prism software version 9.5.0 (GraphPad Software Inc., La Jolla, CA, USA).

## 3 Results

### 3.1 Full-sibling study compared the timing of allergen introduction, parental allergic phenotype, and maternal immunity on the risk of allergy development

We enrolled a cohort of 15 unrelated Icelandic mares and 1 unrelated Icelandic stallion, all living in Iceland with no prior *Cul* allergen exposure. The 15 mares were bred to the same stallion (parents) for three consecutive years, generating three cohorts of full-siblings (C1-C3). The timing of allergen exposure was determined for all horses by the two environments, Iceland or the US, they resided in and is summarized in **Figure 1**. The parents were imported to the US as adults (median 8 years old, range 5–16 years old) when the mares were pregnant with cohort C2 (**Figure 1A**). Full-sibling cohort C1 was previously born in Iceland and was first exposed to allergen in adolescence following import to the US (**Figure 1B**). C2 was born in June when allergen exposure had just started that year (**Figure 1C**). This was also the first allergen introduction for the parents. There was no detectable *Cul*-specific IgE in the colostrum of the dams for C2 (**S1**). The following year, full-sibling cohort C3 was born early summer during allergen exposure (**Figure 1D**). However, the dams had been exposed to allergen the previous summer and developed allergen-specific IgE prior to giving birth to C3 (**S1**). For C3, there was *Cul*-specific colostrum IgE for both major allergens Cul o 2 (median 46.3 RU, range 5–110 RU) and Cul o 3 (median 34.3 RU, range 5–110 RU).

In the US, the parents lived together with similar allergen exposure for at least two years. In the second year, clinical allergy had developed in most of the allergic parents (**Table 1**). C1-C3 also lived in the same *Cul*-endemic environment as their parents, until they were at least young adult age (3.5 years).

*Cul* hypersensitivity is a seasonal allergy with clinical signs occurring during *Cul* allergen exposure during warm humid months (May-October for Ithaca, NY). *Cul* midges do not survive freezing temperatures and, therefore, temperature can serve as a surrogate measurement for midge burden across years. Therefore, the last spring day and the first fall day below freezing marked the beginning and ending of the *Cul* midge exposure season, respectively. For the duration of the study, the number of consecutive days above freezing from May to October each year was 159±9 days (mean±SD) from 2012-2016 (**S2**). This confirms that differences in rate of allergy development were not due to differences in weather, supporting consistent allergen exposure for the duration of the study and while the horses were in Ithaca, NY.

To confirm if any of the individuals had a delayed onset of allergy, a follow-up survey was performed after all horses had at least 9 years of allergen exposure in the US (**S3**). This finalized the rate of allergy development in the parents and each full sibling cohort.

### 3.2 Rate of allergy increased with the age at allergen introduction

**Figure 2:**
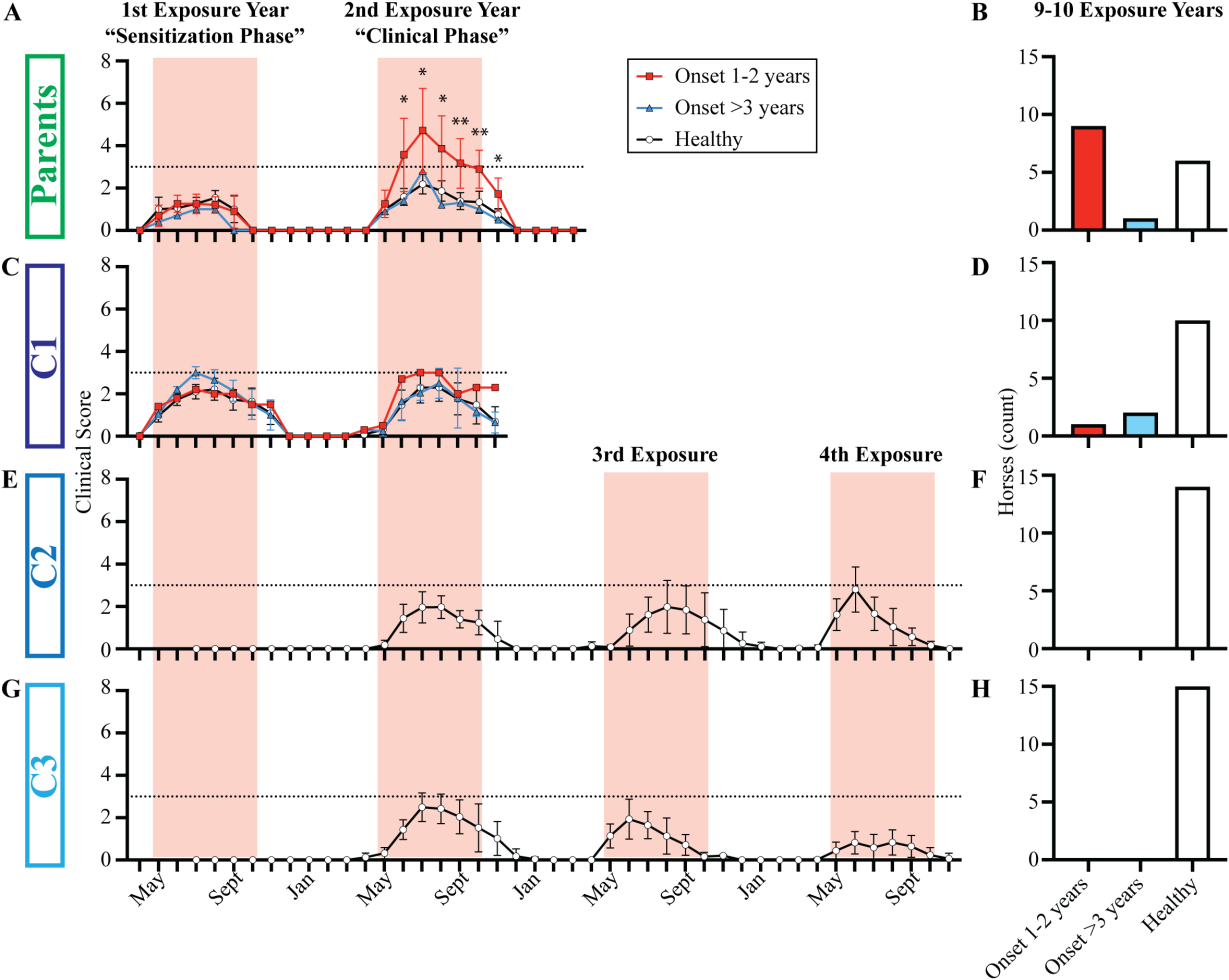
Clinical allergy only developed in individuals with late allergen introduction during adolescence or adulthood. Individuals were categorized as allergic or clinically healthy based on the clinical scoring at Cornell and a follow-up survey performed after either 9 or 10 years of living in allergen endemic environments. Individuals were finally assigned to allergy groups after the 2021 follow-up survey: i) allergy onset after 1–2 years of allergen exposure (red squares), ii) allergy onset after 3+ years of allergen exposure (blue triangles) and iii) healthy individuals (white circles). The timing of allergen exposure was aligned for each group: first summer when allergen sensitization occurred (“Sensitization Phase”), second summer when clinical allergy began in some individuals (“Clinical Phase”), and 3^rd^ and 4^th^ summers when C2 and C3 were monitored until they were 3.5 years old. **(A)** Parent group clinical scores from 2012-2013. **(B)** Parent group allergy status after 10 years of allergen exposure. **(C)** C1 clinical scores from 2013-2014. **(D)** C1 allergy status after 9 years of allergen exposure. **(E)** C2 clinical scores from 2012-2015. Horses were born in June, and scoring began in July of the 1^st^ exposure year. **(F)** C2 allergy status after 10 years of allergen exposure. **(G)** C3 clinical scores from 2013-2014. Horses were born in June, and scoring began in July of the 1^st^ exposure year. **(H)** C3 allergy status after 9 years of allergen exposure. **(A ,C, E, G)** Graphs plot median and range for each group. Horizontal dotted line describes threshold of 3. Pink vertical bars show months with allergen exposure. **(B, D, F, H)** Bar graphs plot number of horses in each group. *p<0.05, **p<0.01.

Upon import to the US, each horse was repeatedly monitored and assigned clinical scores to quantify development of allergy, or lack thereof. In the parent cohort, 62.5% (10 out of 16) developed allergy (**Table 1** **).** None of the parents developed clinical signs in the first year of allergen exposure, during the “Sensitization Phase” (**Figure 2A**, **S4A**). During the second year of allergen exposure, the “Clinical Phase”, 9 horses of the parent cohort developed clinical allergy (**Figure 2A**, **S4B**). After two summers of allergen exposure, some of the parents moved to a different location. However, 5/9 horses with clinical allergy stayed in Ithaca for the entire observation period. The other four allergic parent horses stayed for at least a third year. In this third year, the severity of disease was maintained (**S4C**), and even worsened for some horses. The average highest clinical score for the parent cohort was 6.5±2.2 (mean±SD, **S5**) out of a maximum allergy score of 10. During the follow-up study, one additional mare developed allergy after 6 years of allergen exposure (**Figure 2B**, **S5**). Together, this showed that horses exposed to allergen as adults rapidly developed allergy, which often became severe.

In C1, 21% (3/14) developed allergy (**Table 1**) and 9 of them, including the 3 allergic horses, stayed in Ithaca until the end of the observation period. The rate of allergy in C1 was significantly lower than the rate of allergy in the parent cohort (p = 0.0146). Similar to the parents, none of the horses developed clinical signs during the “Sensitization Phase” (**Figure 2C**, **S4D**). One horse developed clinical allergy during the “Clinical Phase” in the second year of allergen exposure (**Figure 2C**, **S4E**). This individual also worsened in year 3 (**S4F**) and his highest clinical score was 5. During the follow-up study, two additional C1 horses developed clinical allergy after 6 and 7 years of allergen exposure (**S5**). The results of the C1 cohort suggested that earlier allergen introduction decreased the risk of allergy, delayed its onset, and reduced the severity of disease (**Figure 2D**).

**Figure 3:**
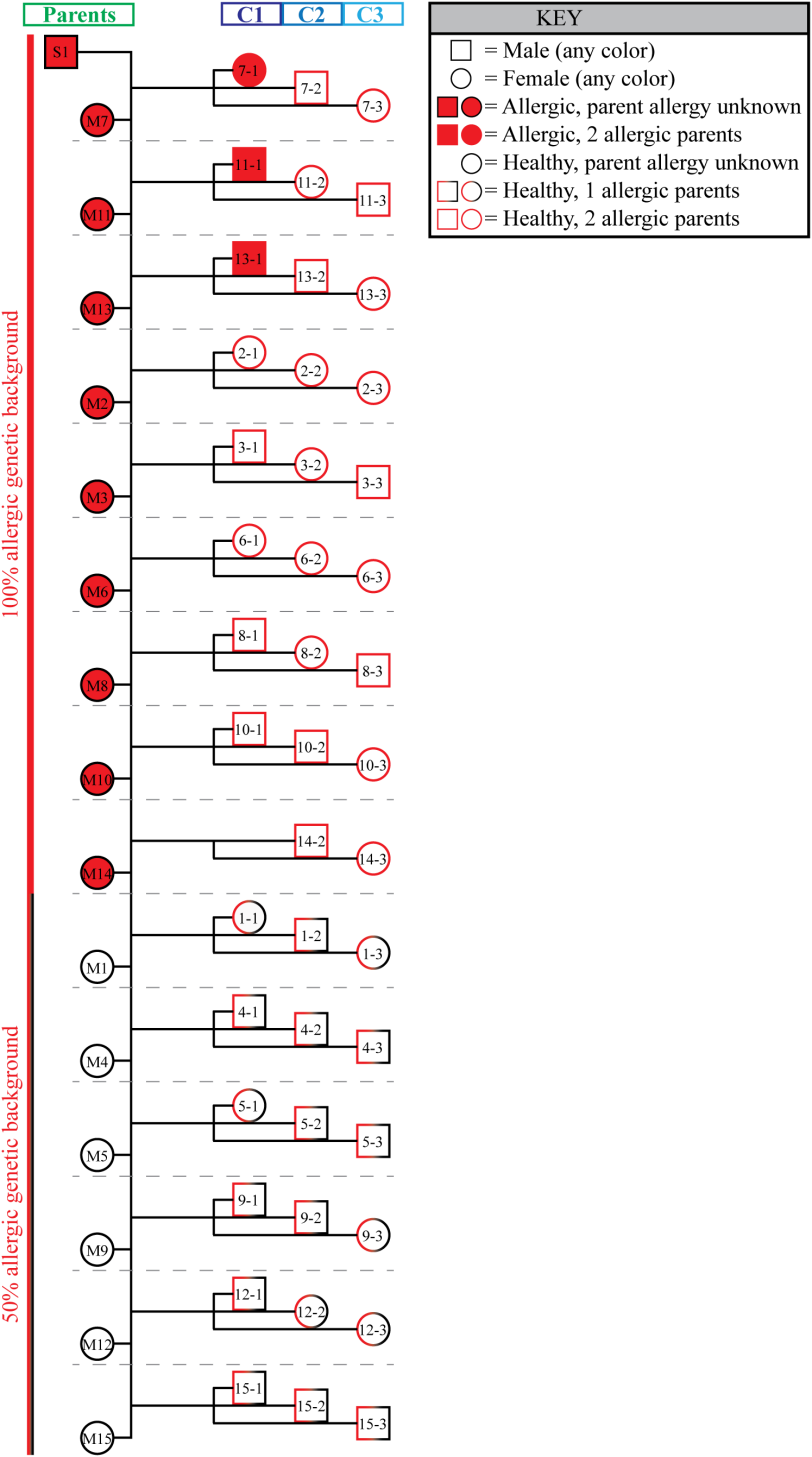
All full-sibling cohorts had at least one allergic parent. A family pedigree summarizes the genetic predisposition to allergy of all three groups. Both male (squares) and female (circles) individuals were included in the study. Individuals that developed allergy are shaded red. The outline of each symbol describes the allergy status of the individual’s parents. All 3 full-sibling cohorts had either 1 parent that developed allergy (half red half black outline; 50% allergic phenotype background), or both parents developed allergy (full red outline; 100% allergic phenotype background). Individual mother/child groups are separated by horizontal gray dotted lines.

In contrast, none of horses in C2 (0/15, **Figures 2E, F**) or C3 (0/15, **Figures 2G, H**) developed allergy (**Table 1**), a significantly lower rate of allergy compared to the parent cohort (p <0.0001 for both C2 and C3). Importantly, the sire developed severe allergy. Thus, either one or both parents of all three full-sibling cohorts were allergic and therefore had an allergic phenotype (**Figure 3**). This suggested that all individuals had an increased genetic risk to develop allergy. However, the differences in the rate of allergy development between the full-sibling cohorts supported that the timing of allergen introduction dominated the clinical outcome and that early allergen exposure downregulated or even eliminated the inherited allergy-associated predisposition.

### 3.3 Early allergen introduction increases allergen tolerance

**Figure 4:**
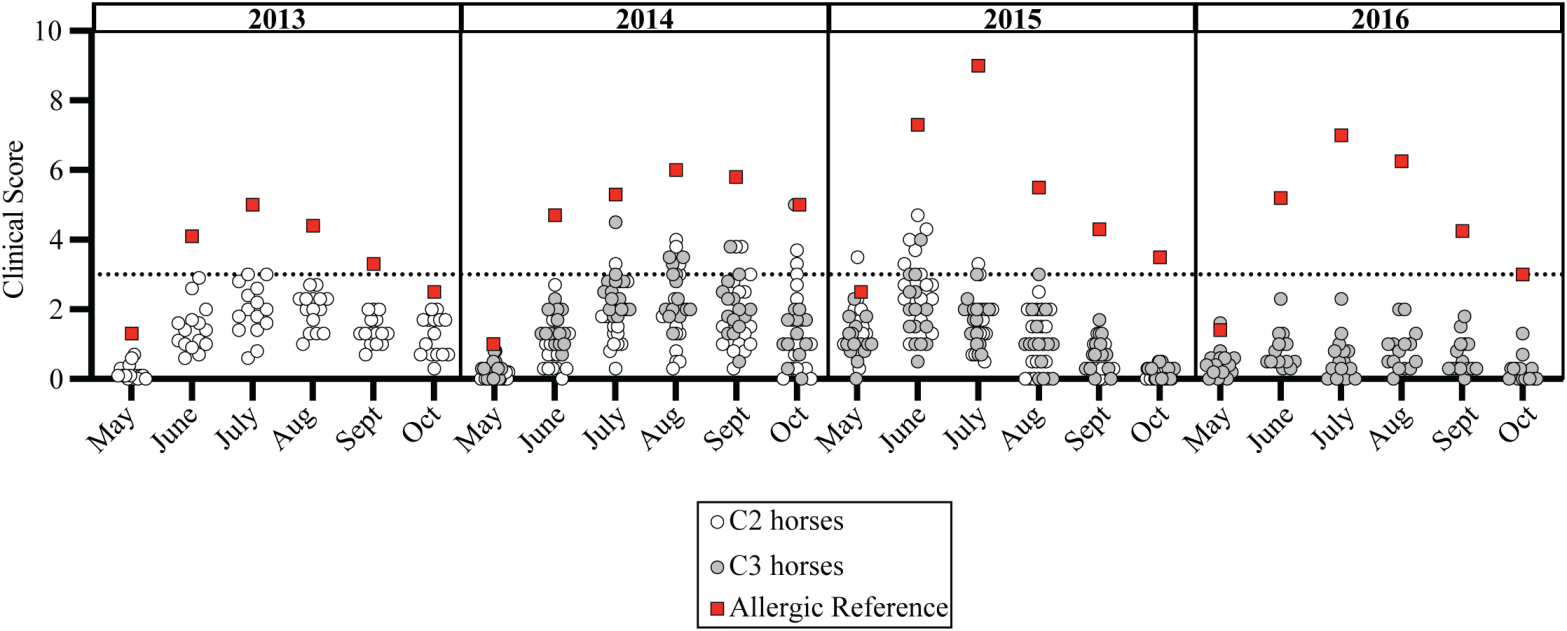
Clinical allergy did not develop in individuals exposed to allergen from birth-on. *Cul* hypersensitivity is a seasonal disease where clinical allergy only develops in summer months during allergen exposure. Monthly average clinical scores from May-October for each C2 horse (open circles, n=15), C3 horse (gray circles, n=14), and an adult allergic indicator horse (red square, n=1) is shown. Clinical scores are shown for allergen exposure at Cornell in year two (2013 for C2, 2014 for C3), three (2014 for C2, 2015 for C3) and four (2015 for C2 and 2016 for C3). All individuals lived together and remained healthy after 9–10 years of allergen exposure. Horizontal dotted line describes threshold of 3.

Both C2 and C3 showed no clinical allergy and scores of 0 during their first year of life with allergen exposure. Interestingly, some individuals in both C2 and C3 had transient clinical scores above the threshold of 3 (**Figure 4**, gray and open circles) in their first 2–4 years of life, but these transient clinical signs did not persist through the summer summer and/or did not recur through subsequent summers (**S6**). These transient scores resulted from mild hair loss and local inflammation at skin areas around midge bites. The clinical presentation clearly contrasted with the severe clinical signs of allergic dermatitis that developed simultaneously in many adult allergic horses of the parent cohort (**Figure 4**, red squares). More specifically, C2 developed these temporary mild skin irritations with average scores at or above 3 during allergen exposure in year two (n=2 in June 2013), three (n=5 in August 2014) and four (n=6 in June 2015) (**S6**). A few C3 horses also had similar skin irritations during allergen exposure years two (n=4 in August 2014) and three (n=3 in June 2015), but not year four (**S6**). None of the C2 or C3 horses developed sustained clinical disease and all remained clinically healthy, as determined during the follow-up survey performed during allergy exposure year 9 or10.

A similar trend also occurred in the healthy horses in C1; during allergen exposure years two (n=2 in July 2014) and three (n=2 in June 2015) (**S4E-F**). In each of these instances for C1, C2 and C3, mild clinical signs of skin irritation resolved quickly, did not last for the duration of the summer, and did not manifest into allergic disease. However, this demonstrated that the healthy horses in C1, C2 and C3 were responding to allergen exposure early in their lives and were able to successfully tolerate *Cul* allergens despite the high allergic pressure, which caused severe clinical signs in their allergic parents living in the same environment.

### 3.4 Maternally-acquired allergen-specific IgE did not increase allergy development

**Figure 5:**
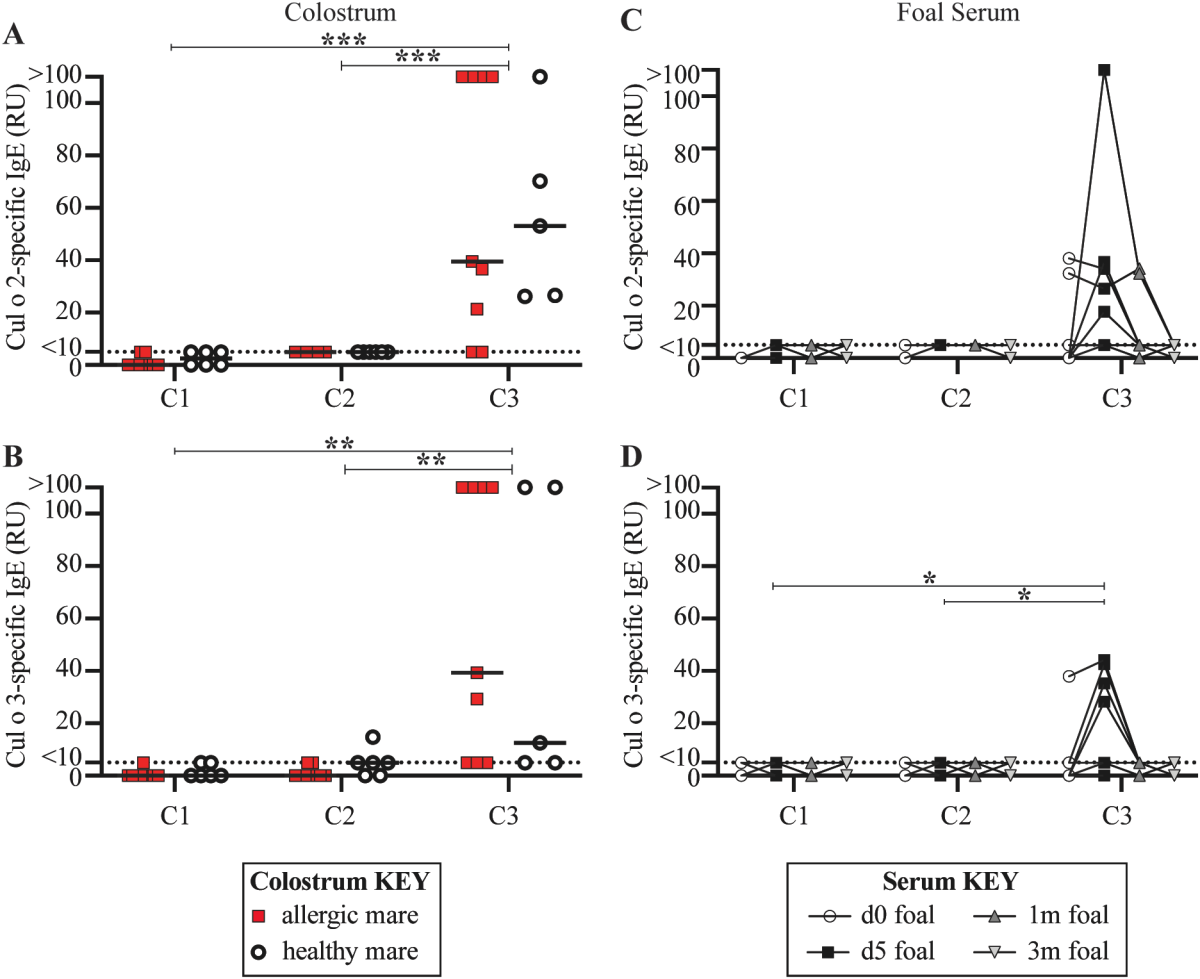
Only C3 received allergen-specific maternal IgE. For each full-sibling cohort (C1-3), allergen-specific IgE was measured in colostrum at birth and in foal serum until foals were 3 months of age. IgE antibodies specific for the two major *Cul* allergens, Cul o 2 and Cul o 3, were compared. Colostrum collected from dams that ultimately developed allergy were retrospectively labeled (allergic dams are red squares and healthy dams are open circles). **(A)** Cul o 2-specific IgE in colostrum. **(B)** Cul o 3-specific IgE in colostrum. Allergen-specific IgE was also measured in foal serum before first suckle (d0, open circles), at 5 days old (d5, black squares), 1 month old (1m, dark gray up triangle) and 3 months old (3m, light gray down triangle). **(C)** Cul o 2-specific IgE in foal serum. **(D)** Cul o 3-specific IgE in foal serum. **(C-D)** A few foals in C3 suckled before their d0 blood draw. Relative Units (RU) are reported for IgE concentrations based on a standard curve run on serum from an adult allergic mare. Relative IgE levels are connected for each individual foal. Asterisks denote statistics comparing combined allergic and healthy C3 mare colostrum with all 15 C1 mare colostrum. **p<0.01, ***p<0.001.

While both C2 and C3 were exposed to allergen from birth-on, only C3 received maternal allergen-specific IgE, while C2 did not. Due to the type of placentation in horses, maternal IgE is exclusively transferred to foals with the colostrum during the first 12–16 hours of life (19, 36). Allergen-specific IgE was measured in the colostrum of each mare directly after foaling and before foals suckled (**Figures 5A, B**, **S1**) and in serum of the foals during the first 3 months of life (**Figures 5C, D**). Foals do not make endogenous IgE until 9–12 months old (36).

There was no detectable Cul o 2-specific IgE (**Figure 5A**, **S1**) or Cul o 3-specific IgE (**Figure 5B**, **S1)** in the colostrum for C1 before the mares left Iceland. As a result, C1 foals did not have any maternally-acquired allergen-specific IgE in their serum in their first 3 months of life (**Figures 5C, D**). This was expected because C1 was born in Iceland and, along with their dams, were naïve to *Cul* allergen.

C2 was born in the US in June 2012, which was during the first month of allergen exposure for the parents. As a result, dams were experiencing their first exposure to *Cul* allergen, the allergy “sensitization phase”, and had not yet developed clinical disease (**Figure 2A**, **S4A**). There was also no detectable Cul o 2-specific IgE (**Figure 5A**, **S1**) or Cul o 3-specific IgE (**Figure 5B**, **S1**) in the colostrum received by C2 foals. Consequently, C2 foals did not have any maternally-acquired allergen-specific IgE in their serum during their first 3 months of life (**Figures 5C, D**).

In contrast, C3 was born in June 2013 when the allergic parents were first developing clinical allergy (**Figure 2A**, **S4B**). In addition to clinical disease, the allergic dams also had elevated colostrum concentrations of Cul o 2-specific IgE (median 39.50 RU, range 5–110 RU, **Figure 5A**, **S1**) and Cul o 3-specific IgE (median 39.30 RU, range 5–110 RU, **Figure 5B**, **S1**). Healthy dams were also exposed to allergen and had Cul o 2-specific IgE (median 53.15 RU, range 26.18–110 RU, **Figure 5A**) or Cul o 3-specific IgE (median 12.5 RU, range 5–110 RU, **Figure 5B**) in their colostrum. Allergen-specific IgE levels in the colostrum of allergic versus healthy mares were similar (p>0.5). There was significantly higher Cul o 2- and Cul o 3-specific IgE in C3 mare colostrum compared to C1 colostrum (p < 0.001 and p = 0.005, respectively) and compared to C2 colostrum (p = 0.001 and p = 0.007, respectively). Accordingly, at 5 days of age, some C3 foals had elevated serum Cul o 2-specific IgE (median 5 RU, range 5–110 RU, **Figure 5C**) and Cul o 3-specific IgE (median 5 RU, range 0-44.16 RU, **Figure 5D**), following colostrum maternal transfer. Serum concentrations of Cul o 2-specific IgE and Cul o 3-specific IgE returned to undetectable levels by 3 months of age (**Figures 5C, D**) due to the lack of endogenous IgE production in foals at this early age. Cul o 3-specific IgE was significantly higher in C3 foal serum at day 5 (d5) compared to C1 foal serum at d5 (p = 0.034) and compared to C2 foal serum at d5 (p = 0.034).

The maternally-acquired allergen-specific IgE by the C3 foals did not increase the rate of clinical allergy development compared to the C2 foals (both 0%), further supporting that the timing of allergen introduction also overrides any predisposition to allergy that may come from maternal transfer of allergen-specific IgE antibodies.

### 3.5 Maternally-acquired allergen-specific IgG as a possible mechanism for increased allergen tolerance

**Figure 6:**
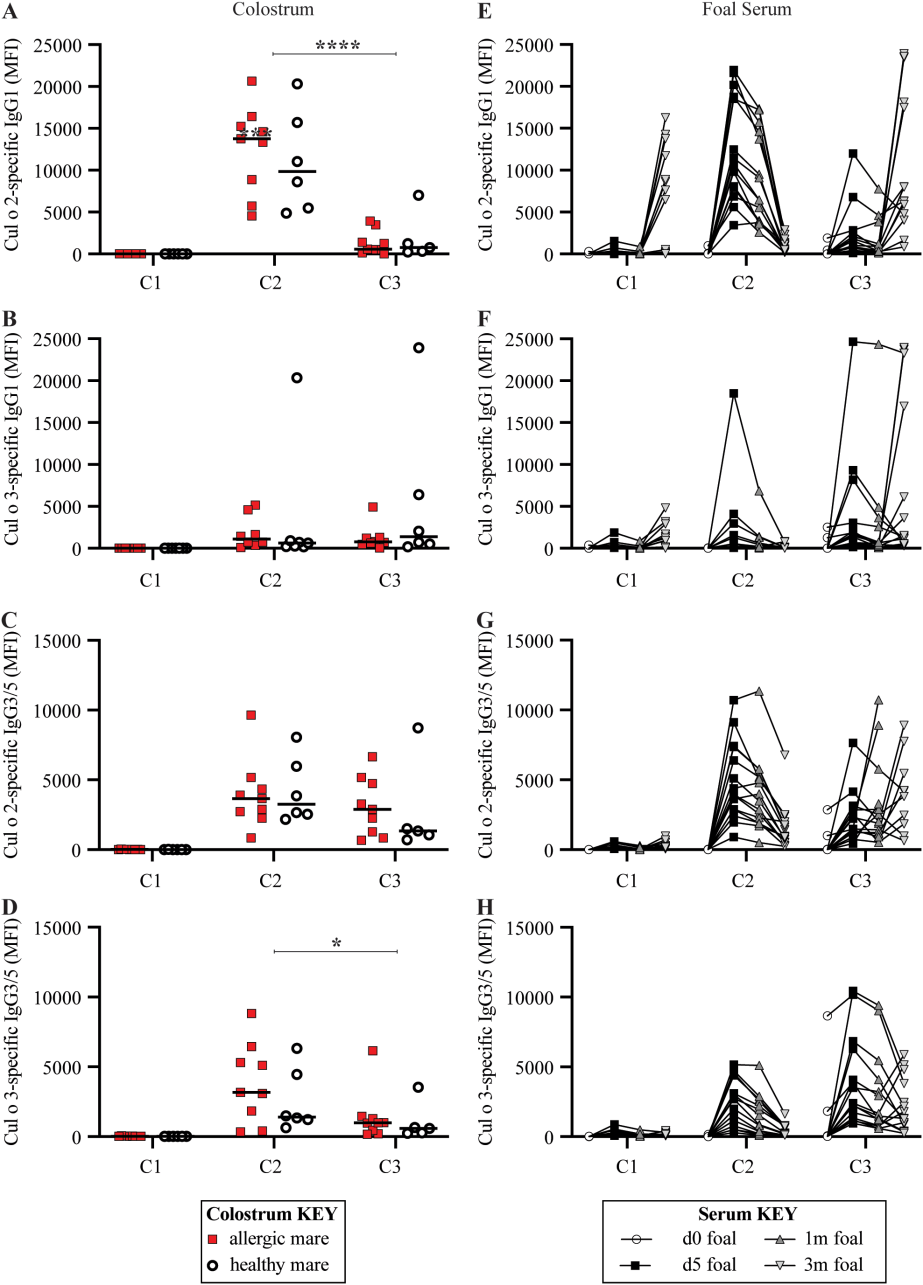
Both C2 and C3 received allergen-specific maternal IgG immunity through colostrum. For each full-sibling cohort (C1-3), allergen-specific IgG1 and IgG3/5 were measured in colostrum at birth and in foal serum until 3 months of life. IgG specific for the two major *Cul* allergens, Cul o 2 and Cul o 3, were compared. **(A–D)** Colostrum collected from dams that ultimately developed allergy are retrospectively labeled (allergic dams are red squares and healthy dams are open circles). **(A)** Cul o 2-specific IgG1 in colostrum. **(B)** Cul o 3-specific IgG1 in colostrum. **(C)** Cul o 2-specific IgG3/5 in colostrum. **(D)** Cul o 3-specific IgG3/5 in colostrum. **(E–H)** Allergen-specific IgG was also measured in foal serum before first suckle (d0, open circles), at 5 days old (d5, black squares), 1 month old (1m, dark gray up triangle) and 3 months old (3m, light gray down triangle). **(E)** Cul o 2-specific IgG1 in foal serum. **(F)** Cul o 3-specific IgG1 in foal serum. **(G)** Cul o 2-specific IgG3/5 in foal serum. **(H)** Cul o 3-specific IgG3/5 in foal serum. **(E–H)** A few foals in C3 suckled before their d0 blood draw. Median fluorescent intensity (MFI) are reported for IgG concentrations. IgG measurements are connected for each individual foal. Asterisks denote statistics comparing combined allergic and healthy C3 mare colostrum with all combined allergic and healthy C2 mare colostrum. *p<0.05, ****p<0.0001.

To further explore the mechanisms underlying the increased allergen tolerance with earlier allergen exposure, we further measured allergen-specific IgG in colostrum and foal serum samples. Horses have seven IgG isotypes (43). IgG3 and IgG5 are both involved in the type 2 immune response that leads to allergy (16, 19, 44). Due to their similar structure in horses, IgG3 and IgG5 are measured together and reported as IgG3/5 (42). IgG1 is the initial IgG isotype in response to many antigens, and is also the first IgG made by foals (45). Allergen-specific IgG1 and IgG3/5 were therefore measured in the colostrum of each mare directly after foaling and before foals suckled (**Figures 6A–D**) and in serum of the foals during the first 3 months of life (**Figures 6E–H**).

There was some detectable Cul o 2- (median 6477 MFI range 5–16273 MFI) and Cul o 3-specific IgG1 (median 890 MFI range 17–4806 MFI) (**Figures 6A, B**) in some of the C1 foals at 3 months of age. This was considered an endogenous response of the C1 foals and likely due to antibodies induced against antigens similar to Cul o 2 present in Iceland. There was no detectable Cul o 2- or Cul o 3-specific IgG3/5 (**Figures 6C, D**) in the colostrum for C1 before the mares left Iceland. Likewise, there was low to no Cul o 2- or Cul o 3-specific IgG1 (**Figures 6E, F**) or IgG3/5 (**Figures 6G, H**) in the foal serum during the first month of life, supporting the lack allergen-specific IgG transfer, allergen exposure, and an immune response to *Cul* in C1. These results were expected because C1 was born in Iceland and, along with their dams, were naïve to *Cul* allergen.

C2 was born in the US during the first season of allergen exposure, the dams’ “Sensitization Phase”. In contrast to allergen-specific IgE, C2 colostrum had detectable Cul o 2-specific IgG1 (median 13357 MFI, range 4555–20660 MFI, **Figure 6A**), Cul o 3-specific IgG1 (median 734 MFI, range 97–20343 MFI, **Figure 6B**), Cul o 2-specific IgG3/5 (median 3661 MFI, range 837–9645 MFI, **Figure 6C**) and Cul o 3-specific IgG3/5 (median 3087 MFI, range 343–8828 MFI, **Figure 6D**). There was no difference in allergen-specific IgG1 levels or IgG3/5 in the colostrum of allergic versus healthy mares (p>0.7). Cul o 2 and Cul o 3-specific IgG1 and IgG3/5 were undetectable in foal serum at birth before suckling. On day 5, Cul o 2-specific IgG1 (median 10823 MFI, range 3417–21947 MFI, **Figure 6E**), Cul o 3-specific IgG1 (median 482 MFI, range 73–18499 MFI, **Figure 6F**), Cul o 2-specific IgG3/5 (median 3958 MFI, range 908–10707 MFI, **Figure 6G**) and Cul o 3-specific IgG3/5 (median 2684 MFI, range 291–5165 MFI, **Figure 6H**) all peaked in foal serum. This supports that C2 received maternally-acquired allergen-specific IgG1 and IgG3/5.

C3 was born in the US during the second season of allergen exposure and the “Clinical Phase”. Cul o 2-specific IgG1 in colostrum for C3 was significantly lower than for C2 (p<0.0001) but Cul o 3-specific IgG1 in colostrum was similar between C2 and C3 (p>0.9). Allergen-specific IgG1 levels in the colostrum of allergic versus healthy mares were similar (p>0.7). Cul o 2-specific IgG1 (median 1018 MFI, range 126–11973 MFI, **Figure 6E**) and Cul o 3-specific IgG1 (median 1474 MFI, range 157–24663 MFI, **Figure 6F**) were also detectable at day 5 in foal serum but were undetectable at birth. Cul o 2-specific IgG3/5 in colostrum for C3 was similar to C2 colostrum levels (p>0.4), and Cul o 3-specific IgG3/5 in colostrum was slightly lower in C3 compared to C2 (p<0.05). Cul o 2-specific IgG3/5 (median 1497 MFI, range 443–7635 MFI, **Figure 6G**) and Cul o 3-specific IgG3/5 (median 2400 MFI, range 953–10443 MFI, **Figure 6H**) were also detectable at day 5 in foal serum, but were undetectable at birth. This supports that C3 also received maternally-acquired allergen-specific IgG1 and IgG3/5. Together, these data support the transfer of maternally-acquired allergen-specific IgG1 and IgG3/5 to C2 and C3 foals, which may have a role in the underlying mechanism of allergen tolerance in foals exposed to allergen from birth-on.

## 4 Discussion

Allergies develop in some individuals following repeated exposure to allergen while others can tolerate allergen exposure. Determining factors that promote allergy development is of great importance in evaluating an individual’s risk of becoming allergic, increasing tolerance to allergens, and ultimately preventing clinical allergy. Genetic predisposition is considered a major contributor to allergy risk, but alone does not fully explain it (1, 7, 46). Maternally-acquired allergen-specific immunoglobulins has also been proposed to contribute to the development, or prevention, of allergies. However, in this study, we have shown strong evidence that early allergen introduction from birth-on overrides the genetic risk likely inherited from parents with an allergic phenotype and the immunological risk of maternal-acquired allergen-specific IgE, and protects the individual from allergy development.

In this unique full-sibling study we compared allergen introduction at different ages, starting at birth, during adolescence, or in adulthood, while individuals stayed in the same environment during allergen sensitization and clinical *Cul* hypersensitivity onset. In the two cohorts exposed to allergen at birth, we also compared the influence of maternal-acquired allergen-specific immunoglobulin. We compared *Cul* hypersensitivity development in three full-sibling cohorts born in three consecutive years to either one or both allergic parents and in two different housing locations, with or without *Cul* allergen exposure. This allowed us to control the onset of allergen exposure in each cohort and the presence or absence of maternally-acquired allergen-specific immunoglobulin in early life. At the time when the parents and C1 horses of this study were housed in Iceland, *Cul* midges were absent from this country. In contrast, *Cul* midges have been endemic in the US since before the start of the study. We observed a drastic reduction in clinical *Cul* hypersensitivity development the younger the individuals were at the time of allergen introduction. Allergy rates of 62.5% were observed in the parents (adult introduction), 21% in C1 (introduction from adolescence-on), and 0% in C2 and C3 (introduction from birth-on). This demonstrated that clinical *Cul* hypersensitivity dramatically decreased with earlier allergen introduction, even if individuals had a high genetic risk (one or both allergic parents with an allergic phenotype), were repeatedly exposed to high environmental allergen loads and were exposed to maternally-acquired allergen-specific IgE and IgG.

*Cul* hypersensitivity is a seasonal, recurrent disease (17, 20, 23–30), where *Cul* are only active in the environment when ambient temperature remains above freezing for extended periods of time. *Cul* allergen exposure is therefore directly related to the length of the warm season from late spring to early fall. To rule out that weather differences year to year may have contributed to the different rates of *Cul* hypersensitivity between cohorts, we compared the average monthly temperatures for the duration of the study. Not only were the general weather trends similar, with the last spring frost in early May and the first fall frost in mid-October, the number of consecutive days above freezing was remarkably similar between years. One severely allergic parent was also included as an “indicator horse” for allergen load in the summers when C2 and C3 were maturing. The consistent allergy severity in this indicator horse demonstrated comparable allergen exposure across all summers, supporting that the difference in allergy rates between cohorts were not due to variations in weather or *Cul* burden.

An interesting difference between cohorts was the timing from allergen introduction to clinical disease onset. As expected with most allergies, the first period of allergen exposure sensitizes the individual, but clinical signs are not yet developing, and the second allergen exposure induces clinical disease (47). The sensitization period is characterized by the formation of allergen-specific IgE antibodies, a process that can take weeks to months. Accordingly, in our model of *Cul* hypersensitivity, the sensitization period for the parents lasted the entire first season of allergen exposure and all but one allergic individual in the adult parent cohort (9/10) developed *Cul* hypersensitivity during their second summer of *Cul* exposure. However, in C1, two of the three horses that became allergic had a delayed onset of *Cul* hypersensitivity development after 6–7 summers of repeated allergen exposure. Severe allergic disease, characterized by recurrent, persistent high clinical scores, was also more frequently seen in horses with early *Cul* hypersensitivity onset during the 2^nd^ or 3^rd^ year of allergen exposure. In general, allergic horses in the parent cohort more often developed severe allergy compared to the allergic horses in C1. Together, this suggests that early allergen introduction not only reduces the risk of *Cul* hypersensitivity development, but also delays the onset of clinical *Cul* hypersensitivity and decreases the severity of clinical disease in those individuals that will still develop clinical *Cul* hypersensitivity. In conclusion, our uniquely controlled allergen exposure model strongly supports the hypothesis (48, 49) that early-in-life allergen exposure from birth-on prevents *Cul* hypersensitivity development.

The most likely explanation for the absence of clinical *Cul* hypersensitivity in C2 and C3 is an increase of immunological tolerance to *Cul* allergen induced by the exposure from birth-on. In addition, both C2 (n=10) and C3 (n=6) included horses with transient scores over 3. However, these elevated scores and clinical signs did not persist throughout the entire season of allergen exposure, and did not recur from year to year during the study. As a result, these horses were considered non-allergic, as *Cul* hypersensitivity is both a persistent and recurrent disease. These transient scores support that C2 and C3 were exposed to and were immunologically responding to *Cul* midges, but were able to develop immune tolerance and prevent *Cul* hypersensitivity development.

There are several mechanistic explanations that could explain this increase in tolerance development: First, epigenetic modifications to DNA and histones near promoter regions influence gene expression, increase with age, and are altered based on environmental exposure (50). As a result, epigenetic modifications can bridge environmental influences on gene expression (51). Horses that are exposed early-in-life to allergen might experience a different epigenetic programming that prevents the development of *Cul* hypersensitivity. In fact, methylation profiles have been recently used to discriminate between allergic and healthy children (52). DNA methylation patterns have been found to predict clinical outcomes of egg and peanut allergy (53). Children with food allergy also have epigenetic dysregulation in CD4^+^ T cell activation genes (54), further supporting a role in the development of, or protection from, allergies. Here, we hypothesize that the timing of allergen introduction can change the epigenetic landscape in an individual to protect against *Cul* hypersensitivity development.

Second, the maturity of the immune system changes with age (55), which may further explain tolerant versus allergic responses to allergens at different introduction times. A recent study found that early peanut introduction increased allergen-specific IgG4, and not IgE, even in children with an allergy-associated HLA allele (56). Compared to allergen-tolerant infants, infants with food allergies have an overall increase in activated B cells (57), increased allergen-specific CD4^+^ T cells (58), and inflammatory monocytes (59). We therefore propose that the stage of immune cell development during the exact moment of allergen introduction will greatly influence the resultant immune response.

Whether differences in epigenetic modifications, immune tolerance development, other underlying processes, or several of these mechanisms together cause the decrease in clinical *Cul* hypersensitivity when individuals are exposed to allergen early-in-life still needs to be explored in detail to better understand the prominent regulatory routes preventing IgE-mediated allergies.

Our study also sought to explore the role of maternally-derived allergen-specific IgE and IgG on *Cul* hypersensitivity development. In humans, allergen-specific IgE can cross the placenta through IgE-IgG complexes with FcRn, can bind IgE-receptors on fetal mast cells, and has the potential to enable early allergen responses in infants, increase the allergic environment, and increase the risk of allergy development (60). We therefore examined the role of maternally-acquired allergen-specific IgE on allergy development in our study. Horses do not have placental-transfer of immunoglobulins and foals do not produce endogenous IgE antibodies in the first months of life (36). Colostrum, or “first milk”, is secreted for the 12–24 hours after birth (58). Even though both human and equine colostrum contain high concentrations of antibodies, horses do not have placental antibody transfer *in utero* and, therefore, all maternally-acquired immunity in horses is exclusively derived from the colostrum (61). This includes any allergen-specific IgE and IgG antibodies that are present in the colostrum at birth. IgE and IgG can be transferred to the foal’s circulation within the first 12–16 hours of life before the neonatal gut closes for antibody transfer (36). We included three equine immunoglobulin isotypes in this study due to their relevance in early equine immunity and in allergy. IgG1is the IgG isotype that is initially produced by horses in response to most antigens and pathogens including the early immune response to *Cul* allergens (45, 62). IgG3/5 is the equine isotype that we have previously found to be most associated with *Cul* hypersensitivity. Specifically, Cul o 2-specific IgG3/5 is produced in horses before they develop clinical disease (16). Equine IgG3/5 is also predicted to precede IgE, which sequentially class-switches to IgE through IgG precursors (63, 64). IgE is the allergy effector isotype which binds to IgE receptors on mast cells, basophils and some monocytes to induce clinical signs of allergy. We measured IgE, IgG1 and IgG3/5 specific for the two *Cul* allergens, Cul o 2 and Cul o 3, that are most prominent in the NorthEastern United States where the horses lived for the duration of the study (16).

C2 and C3 were the only cohorts born to dams that had been exposed to *Cul* allergen before or during conception, gestation and birth, and therefore had allergen-specific IgE, IgG1 and/or IgG3/5. In this study, the dams in the parent cohort had neither allergen-specific IgE during the birth of C1, because they resided in Iceland, or during the birth of C2, because their birth coincided with the same month when the dams were first exposed to *Cul* allergen and allergen-specific IgE secreting cells had not yet developed (47). Consequently, the colostrum of the dams when C2 was born did not have any allergen-specific IgE antibodies, but did have allergen-specific IgG1 and IgG3/5, which suggested the early rapid response to *Cul* allergen. However, before birth of C3, dams had been exposed to *Cul* allergen in the previous summer and had allergen-specific IgE in their colostrum which was transferred to their neonatal foals. We have previously shown that IgE is rapidly produced in allergen-sensitized individuals following allergen exposure and the IgE increase precedes clinical signs typically by 3–4 weeks (64). We have also shown that both allergic and healthy horses can be sensitized to allergen including production of some allergen-specific IgE (14), explaining why C3 foals born to both healthy and allergic dams still received allergen-specific IgE. Therefore, C3 received allergen-specific IgE and some allergen-specific IgG1 and IgG3/5 at birth. We propose that the slightly lower levels of allergen-specific IgG1 and IgG3/5 in the colostrum of C3 may be due to the development of allergen tolerance in healthy dams and class switching to IgE in allergic dams. Despite the difference in maternal allergen-specific IgE transfer between C2 or C3 or the observation that almost all C3 foals received maternal allergen-specific IgE, none of these horses developed *Cul* hypersensitivity. This supported the conclusion that maternal IgE transfer did neither change the risk of *Cul* hypersensitivity development nor did it influence the strong protective effects of early allergen introduction to reduce clinical *Cul* hypersensitivity.

IgE class switched B cells develop almost exclusively from IgG intermediates (65). In contrast to the slowly developing IgE response, IgG antibodies can typically be detected in the circulation of horses within 6–8 days after antigen challenge, e.g. after viral infection (62, 66). Here, the dams were exposed to allergen during the same month as the birth of C2. It was thus expected that they were experiencing an initial allergen-induced immune response and class switching to IgG, even though it was too early for allergen-specific IgE development. This was confirmed by the presence of allergen-specific IgG1 and IgG3/5 antibodies in the dams’ colostrum in their first and second summer of *Culicoides* exposure, an immune response that was passively transferred to their C2 and C3 foals. By the birth of C3, some of the allergen-specific IgG^+^ B cells likely had switched to IgE or remained IgG^+^ to promote allergen tolerance.

While human studies also suggest many benefits for early allergen introduction, there remain many confounding factors inherent to human studies. These confounding factors likely explain why different studies have come to different conclusions on the impact of early allergen introduction (5, 56, 67–70). Human cohorts typically live in different locations with a range of varying environmental exposures, including household and air pollution exposures that can alter the immune response and increase the risk of allergy development (71). Together these confounding factors beg for the use of a model system that can explore the role of allergen introduction alone, without other environmental barriers. We would like to acknowledge that the horses that moved to live with private owners may may have been misclassified or not identified as allergic if they were only mildly affected. To prevent against this potential bias, all horses lived on the same farm together for at least four years to be consistently monitored before moving to private farms. We also asked for follow-up with any owner who was unsure if their horse had developed clinical signs, to help eliminate any missed disease.

In conclusion, we present evidence that introduction of allergen early-in-life is the greatest protector against *Cul* hypersensitivity development even if born to parents with an allergic phenotype or in the presence of maternally-acquired IgE or IgG. Using a full-sibling model of Icelandic horses, we compared timing of first allergen exposure, while controlling for all other factors – maternal diet, offspring diet, frequency, and duration of allergen exposure, as well as the environment and any other associated exposures, and in the absence of any medical intervention. The related cohorts that were exposed to allergen (i) as adults rapidly developed *Cul* hypersensitivity, (ii) during adolescence developed *Cul* hypersensitivity eventually with lower frequency and disease severity, and (iii) from birth-on, regardless of allergic or healthy parents, did not develop *Cul* hypersensitivity. In addition, the complete protection protection from *Cul* hypersensitivity development in both cohorts exposed from birth-on was independent from exposure to maternally-acquired allergen-specific IgG and IgE. These findings strongly demonstrate that early allergen introduction from birth-on overrides *Cul* hypersensitivity predisposition and prevents clinical *Cul* hypersensitivity development.

## Data Availability

All original data presented in this study are included in the **Supplementary Material**. Further inquiries can be directed to the corresponding author.
