# Supplementary material for: Early allergen introduction overrides allergy predisposition in offspring of horses with Culicoides hypersensitivity
Source: Front Immunol. 2025 Oct 21;16:1654693. doi: 10.3389/fimmu.2025.1654693 (PMC12583015; doi:10.3389/fimmu.2025.1654693)
Supplement: Supplementary file 1 [file DataSheet1.pdf]

Supplementary Material

1 Supplementary Figures and Tables

S1: Allergen-specific IgE in the dam's colostrum was detected by ELISA for each year that full-sibling cohorts 1-3 (C1-3) were born.

	Cul o 2-specific IgE † Median (range), relative units		Cul o 3-specific IgE † Median (range), relative units	
Cohort	Allergic Dam	Healthy Dam	Allergic Dam	Healthy Dam
C1	0 (0-5)	2.5(0-5)	0 (0-5)	0 (0-5)
C2	5 (5-5)	5 (5-5)	0 (0-5)	5 (0-14.76)
C3	39.50 (5-110)	53.15 (26.18-110)	39.3 (5-110)	12.5 (5-110)

† The *Culicoides* allergens Cul o 2 and Cul o 3 were coated to the ELISA plates. The ELISA detection range was 10-100 RU. Absorbance values below the plate blank were considered 0 RU. Absorbance values below the lowest standard value were considered 5 RU. Absorbance values above the maximum standard curve and are reported as 110 RU.

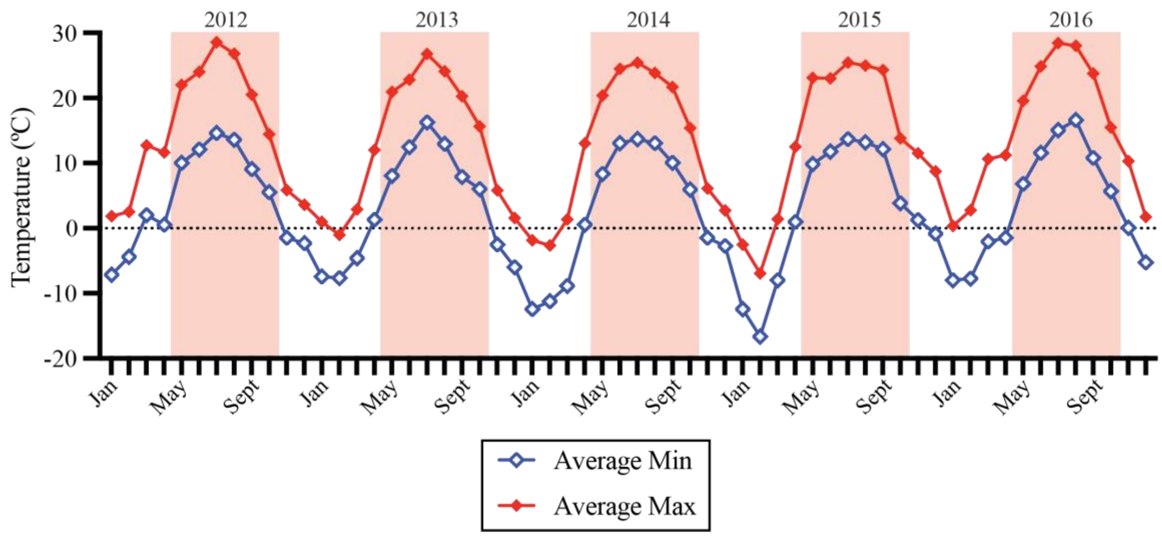

S2: Summer temperatures were consistent for the duration of the study, supporting comparable allergen exposure each year.

The average monthly minimum (blue open diamonds) and maximum (red closed diamonds) temperatures from January 2012 – December 2016, the entire study duration, are summarized. The dotted horizontal line is at 0ºC. Vertical pink shading shows timeframes of consistent temperatures above freezing (May – October), indicative of the *Cul* midge season.

Cornell Icelandic Horse Follow-Up Survey<br>Please fill out the below survey for each Icelandic horse purchased from Cornell University.
1. Horse Name?<br>2. Where does your horse currently live (state/country)?<br>3. How is your horse housed?<br>a. Primarily inside<br>b. Primarily outside<br>c. Split between outside and inside<br>d. Other:<br>4. Has your horse ever experienced any of the following signs of summer eczema (also known as sweet itch, summer itch, or equine dermatitis)? Please refer to the reference sheet on pages 3-5.<br>a. Hair loss<br>b. Persistent itchiness<br>c. Inflamed or crusty skin<br>d. Skin wounds<br>e. I have never observed any of these signs on my horse.<br>f. I don't know<br>g. Other:
5. If you have observed any of the above signs (hair loss, persistent itchiness, inflamed or crusty skin, and/or skin wounds), are they seasonal and reoccur each year?<br>a. Yes, these signs are seasonal and reoccur every year.<br>b. No, I observe these signs all year round.<br>c. I have never observed any of these signs on my horse.<br>d. Other:<br>6. How old was your horse when you first observed signs of summer eczema?<br>7. When do signs of summer eczema begin to appear each year?<br>8. When do signs of summer eczema typically resolve each year?<br>9. Does your horse experience summer eczema every year?<br>a. Yes<br>b. No

S3: Owner follow-up survey questions to assess allergy status of all cohorts.
Questions 1-4 were asked for all horses. Questions 5-9 were only asked if the horse had any of the reported clinical signs in question 4. Pictures of allergic horses and affected skin locations were provided with the survey to help owners identify similar skin alterations in their horses.

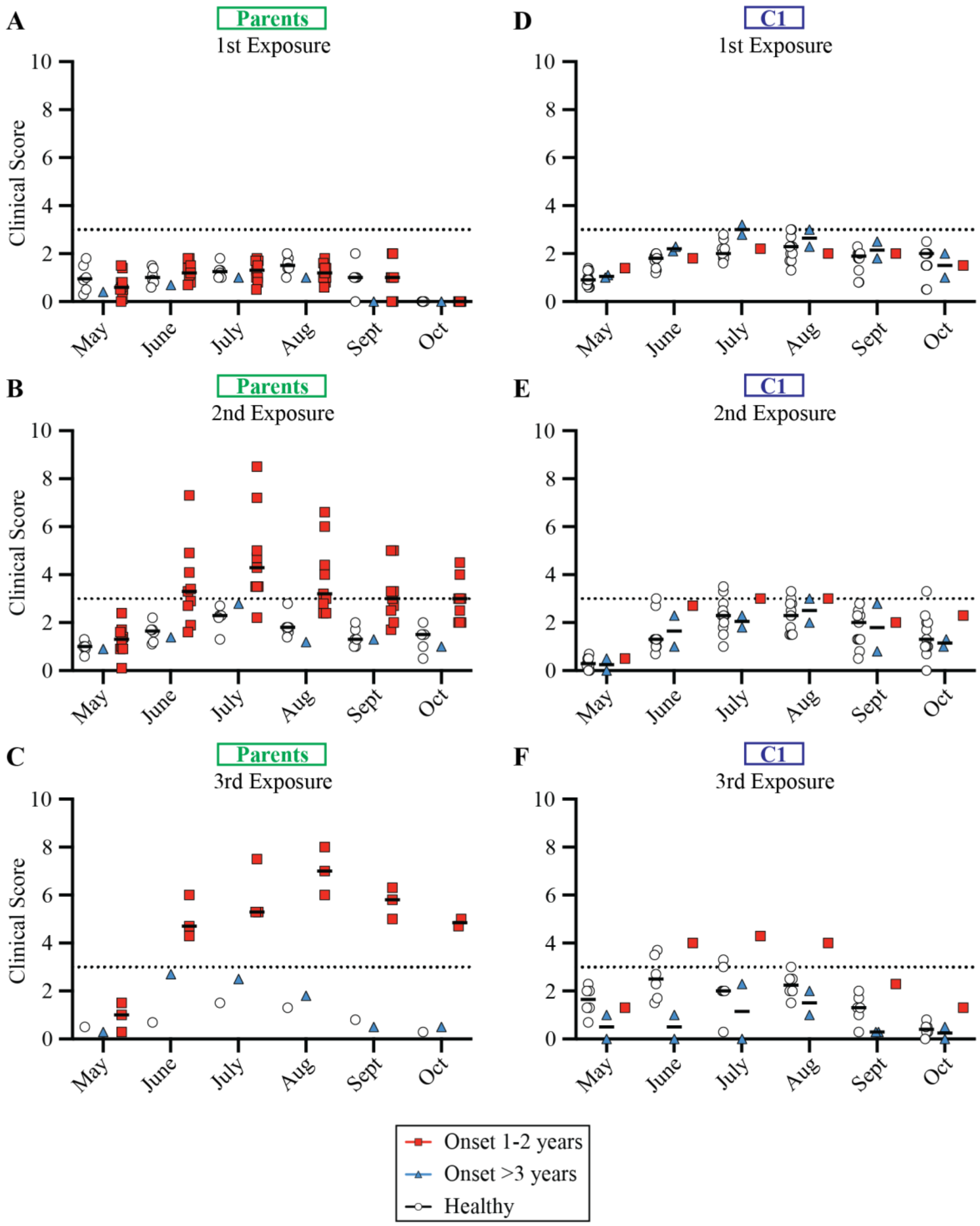

S4: Severe clinical allergy developed rapidly in horses with delayed allergen introduction in adulthood.

Monthly average clinical scores for each parent and C1 horse are shown during the first three summers of allergen exposure. All individuals lived together at Cornell. Individuals were assigned to allergy groups retrospectively: i) allergy onset after 1-2 years of allergen exposure (red squares), ii) allergy onset after 3+ years of allergen exposure (blue triangles) and iii) healthy individuals (white

circles). The timing of allergen exposure was aligned for each group: first summer when allergen sensitization occurred ("Sensitization Phase"), second summer when clinical allergy began in some individuals ("Clinical Phase"). A) Parent group clinical scores from May-October 2012. B) Parent group clinical scores from May-October 2013. C) Parent group clinical scores from May-October 2014. Only 4 allergic and 1 healthy horse were still living at Cornell for this summer. D) C1 clinical scores from May-October 2013. E) C1 clinical scores from May-October 2014. F) C1 clinical scores from May-October 2015. Graphs plot average monthly score for each individual and group median. Horizontal dotted line describes allergy scoring threshold of 3.

S5 : Clinical allergy scores, age of allergen introduction, and disease onset in each individual.

<table>
<tr><th>Cohort</th><th>Horse</th><th>Age at allergen introduction (years) †</th><th>Allergy severity (Highest Score) ‡</th><th>Years of allergen exposure before onset (years) §</th><th>MHC class I haplotype ¶</th></tr>
<tr><td>Parent</td><td>Stallion</td><td>16</td><td>10</td><td>1</td><td>1 / 2</td></tr>
<tr><td>Parent</td><td>Mare 7</td><td>11</td><td>3</td><td>6</td><td>11 / 12</td></tr>
<tr><td>Parent</td><td>Mare 14</td><td>10</td><td>10</td><td>1</td><td>9 / 23</td></tr>
<tr><td>Parent</td><td>Mare 13</td><td>9</td><td>7</td><td>1</td><td>3 / 22</td></tr>
<tr><td>Parent</td><td>Mare 6</td><td>9</td><td>6</td><td>1</td><td>9 / 10</td></tr>
<tr><td>Parent</td><td>Mare 11</td><td>8</td><td>8</td><td>1</td><td>5 / 19</td></tr>
<tr><td>Parent</td><td>Mare 10</td><td>8</td><td>6</td><td>1</td><td>17 / 18</td></tr>
<tr><td>Parent</td><td>Mare 2</td><td>7</td><td>4</td><td>1</td><td>5 / 6</td></tr>
<tr><td>Parent</td><td>Mare 8</td><td>6</td><td>5</td><td>1</td><td>13 / 14</td></tr>
<tr><td>Parent</td><td>Mare 3</td><td>5</td><td>5</td><td>1</td><td>2 / 4</td></tr>
<tr><td>C1</td><td>Foal 11-1</td><td>2</td><td>5</td><td>2</td><td>2 / 5</td></tr>
<tr><td>C1</td><td>Foal 13-1</td><td>2</td><td>4</td><td>6</td><td>1 / 3</td></tr>
<tr><td>C1</td><td>Foal 7-1</td><td>2</td><td>3</td><td>7</td><td>2 / 11</td></tr>
<tr><td>Healthy Parents, n=6</td><td></td><td>≥5</td><td rowspan="4" colspan="2">Not allergic</td><td rowspan="4">See footnote 4.</td></tr>
<tr><td>Healthy C1, n=11</td><td></td><td>2</td></tr>
<tr><td>Healthy C2, n=15</td><td></td><td>At birth</td></tr>
<tr><td>Healthy C3, n=15</td><td></td><td>At birth</td></tr>
</table>

† Horses in puberty at 2 years of age, and adults at 3 years of age. Horses are arranged by age at allergen introduction, from oldest to youngest.

‡ The highest allergy score, out of a total score of 10, is reported for each allergic horse while they still lived in the same environment.

§ Allergy development first occurs in a silent sensitization phase, followed by the development of clinical allergy. As a result, all horses in this study experienced at least 1 year of allergen exposure before developing clinical disease. Clinical allergy onset in the second year (or later) is commonly observed for *Cul* hypersensitivity.

¶ Horses in this study had diverse MHC class I haplotypes. MHC I haplotype acquired from sire (left number) and dam (right number) are shown for each allergic horse. The healthy horses had a variety of MHC haplotypes, sharing individual haplotypes with the allergic horses. Occasionally, 2-3 healthy horses from different cohorts had identical haplotypes. MHC haplotypes for all horses were measured and published previously [41].

S6: Transiently elevated monthly clinical scores in C2 and C3 were ultimately not persistent or recurrent.

Transient Score Outcome †	Cohort (n=) ‡	Highest score	Longest period with score >3 (months)	Number of years of recurrence §
Not Persistent and Not Recurrent	C2 (n=3)<br>C3 (n=1)	4.7<br>4	1<br>1	0<br>0
Persistent but Not Recurrent	C2 (n=3)<br>C3 (n=2)	4<br>3.3	2-4<br>2-4	0<br>0
Not Persistent but Recurrent	C2 (n=4)<br>C3 (n=3)	4.3<br>5	1-3<br>1-5	1-2<br>1

† For this longitudinal study, clinical signs of *Cul* hypersensitivity were evaluated by repeated clinical allergy scoring. For each month and horse, all scores were averaged as a monthly score. For some of the young horses in C2 and C3, clinical signs were mild, typically occurred early summer with *Cul* exposure, and/or did not continue during heavy *Cul* exposure later in summer/fall. Clinical scores are categorized by their persistence and recurrence. "Not persistent and not recurrent" means that these horses had an average monthly clinical score >3 for only one month during only one year of their life. "Persistent but not recurrent" means that these horses had persistent scores >3 for 2-4 months in a row but for only one year. "Not persistent but recurrent" means that these horses had scores >3 for 1-5 months for 1-2 subsequent years but no scores >3 at any point after that until the end of the study's observation period.

‡ The sample size is the number of horses in the corresponding cohort who had transient clinical signs and scores >3.

§ After the years of recurrence with clinical scores >3, these horses never exhibited clinical signs or clinical scores >3 and were considered non-allergic.

S7 : Datasets used for this study.

Excel file is uploaded separately.
